# Herbal medicine (Bojungikki-tang) for allergic rhinitis: A protocol for a systematic review of controlled trials

**DOI:** 10.1097/MD.0000000000009551

**Published:** 2018-01-19

**Authors:** Ju Ah Lee, Soobin Jang, Ji Hee Jun, Myeong Soo Lee, Eunhee Lee, Namkwen Kim, Dong Hyo Lee

**Affiliations:** a Department of Korean Internal Medicine, College of Korean Medicine, Gachon University, Gyeonggi-do; b KM Fundamental Research Division; c Clinical Research Division, Korea Institute of Oriental Medicine, Daejeon; d Department of Obstetrics and Gynecology, College of Korean Medicine, Woosuk University, Jeonju; e Department of Ophthalmology and Otolaryngology and Dermatology, School of Korean Medicine, Pusan National University, Yangsan; f Department of Ophthalmology, Otolaryngology and Dermatology, College of Korean Medicine, Woosuk University, Jeonju, Korea.

**Keywords:** allergic rhinitis, Bojungikki-tang, Bu-Zhong-Yi-Qi-Tang, herbal medicine, Hochu-ekki-to, protocol, systematic review

## Abstract

**Background:**

Bojungikki-tang, a herbal medicine for strengthening the digestive system, has been widely used in East Asian countries since the Yuan Dynasty in China. Moreover, Bojungikki-tang has reportedly exhibited anti-allergy effects in many clinical trials. This systematic review will assess the effectiveness and safety of Bojungikki-tang for allergic rhinitis (AR).

**Methods and analysis:**

Eleven databases, including Asian databases, will be searched for studies conducted through October 2017. We will include randomized controlled trials (RCTs) assessing Bojungikki-tang for AR. The risk of bias will be evaluated using the Cochrane risk of bias assessment tool, and confidence in the cumulative evidence will be evaluated using the Grading of Recommendations Assessment, Development, and Evaluation (GRADE) instrument.

**Ethics and dissemination:**

This systematic review will be published in a peer-reviewed journal and will also be disseminated electronically and in print. The review will be updated to inform and guide health care practices.

**Registration:**

The protocol for this systematic review has been registered in PROSPERO (https://www.crd.york.ac.uk/PROSPERO) under number CRD42017068993.

## Abstract 2

Supplemental Digital Content is available in the text

## 1 Introduction

Allergic rhinitis (AR) is a symptomatic disorder that is induced after allergen exposure via IgE-mediated inflammation of nasal membranes. The major symptoms of AR are sneezing, rhinorrhea, nasal itching, and nasal obstruction; these symptoms are often accompanied by nasal mucosal swelling, eye itching, cough, postnasal drip, and fatigue from nasal discomfort.^[1]^ Approximately 15% to 20% of the global population is affected by AR, and the prevalence of AR tends to be higher in Westernized countries than in other nations.^[2,3]^ The treatment of AR is focused on alleviating symptoms and removing difficulties associated with daily life. First, patients with AR should avoid allergens such as pollen, dust mites, pet dander, and cockroach infestations. Oral or intranasal antihistamines, intranasal cromolyn, and intranasal corticosteroids are first considered to reduce inflammation if allergic symptoms persist; in certain cases, surgical treatment is recommended.^[4]^

Recently, the use of complementary and alternative medicine (CAM) for treating AR has been increasing worldwide.^[5,6]^ Many prior trials have evaluated the effectiveness of acupuncture,^[7,8]^ aromatherapy,^[9]^ and herbal medicines for AR.^[10,11]^ An analysis of electronic medical records indicated that Bojungikki-tang was the second most prescribed insurance-covered herbal medicine for AR at 3 Korean medical university hospitals.^[12]^

Therefore, this study will seek to systematically review randomized controlled trials (RCTs) to assess the effectiveness and safety of Bojungikki-tang for the treatment of AR. This protocol will describe search methods that involve Asian databases and will therefore permit the inclusion of CAM-related literature that cannot be retrieved from English-language databases.

## 2 Methods

### 2.1 Study registration

This study will follow the guidelines outlined in the Preferred Reporting Items for Systematic Reviews and Meta-Analysis (PRISMA) statement for meta-analyses of health care interventions^[13]^; in addition, the current protocol report adheres to the PRISMA Protocols (PRISMA-P).^[14]^ The protocol for this systematic review has been registered in PROSPERO (https://www.crd.york.ac.uk/PROSPERO) under number CRD42017068993.

### 2.2 Data sources

The following databases will be searched from inception to the present date: MEDLINE, Embase, the Cochrane Central Register of Controlled Trials (CENTRAL), AMED, and CINAHL. We will also search 6 Korean medical databases (OASIS, the Korean Traditional Knowledge Portal, the Korean Studies Information Service System, KoreaMed, the Korean Medical Database, and DBPIA) and 3 Chinese databases: CNKI (including the China Academic Journal, the China Doctoral Dissertations and Master's Theses Full-text Database, the China Proceedings of Conference Full-Text Database, and the Century Journal Project), Wanfang and VIP. In addition, we will search a Japanese database and conduct nonelectronic searches of conference proceedings, our own article files, and 9 traditional Korean medicine journals. The search strategy that will be applied to the MEDLINE database is presented in Supplement 1. Similar search strategies will be used for the other databases.

## 3 Types of studies

Prospective RCTs that evaluate the effectiveness of Bojungikki-tang for AR will be included in this review. Both treatment with Bojungikki-tang alone and concurrent treatment with Bojungikki-tang and another therapy will be considered acceptable if Bojungikki-tang is applied to the intervention group only and any other treatment is provided equally to both groups. Trials with any type of control intervention will be included. There will be no restrictions on publication language. Hard copies of all articles will be obtained and read in full.

## 4 Types of participants

All types of AR patients, regardless of nasal surgery history, will be eligible for inclusion. Participants who have both AR and accompanying diseases will be excluded. There will be no restrictions based on other conditions, such as age, sex, or symptom severity.

## 5 Types of interventions

Interventions of any formulation (i.e., decoction, tablet, pill, powder, and/or nasal spray) of Bojungikki-tang will be eligible for inclusion. The compositions of interventions will be reviewed, and interventions involving herbal combinations that differ from original Bojungikki-tang from the perspective of traditional East Asian medicine will be excluded from this review.

## 6 Data extraction and quality assessment

Hard copies of all articles will be obtained and read in full. Two authors (JAL and SJ) will perform the data extraction and quality assessment using a predefined data extraction form. In addition, all interventions that involve acupuncture will be extracted using the Standards for Reporting Interventions in Clinical Trials of Acupuncture (STRICTA). The risk of bias will be evaluated using the Cochrane risk of bias assessment tool, version 5.1.0, which considers random sequence generation, allocation concealment, blinding of participants and personnel, blinding of outcome assessment, completeness of outcome data, selective reporting, and other sources of bias.^[15]^ The results of such evaluations will be presented by utilizing scores of “L,” “U,” and “H” to indicate a low risk of bias, an uncertain risk of bias, and a high risk of bias, respectively. Disagreements will be resolved by discussion among all authors. When disagreements regarding selection cannot be resolved through discussion, an arbiter (DHL) will make the final decision.

## 7 Data collection and synthesis

### 7.1 Outcome measures

#### 7.1.1 Primary outcomes

The primary outcomes will be the therapeutic effects of treatment on AR.

#### 7.1.2 Secondary outcomes

The secondary outcomes will include safety, which will be evaluated based on adverse effects. Improvements in symptoms (e.g., sneezing, rhinorrhea, nasal itching, and nasal obstruction) will be included as secondary outcomes.

### 7.2 Assessment of bias in the included studies

We will independently assess bias in the included studies in accordance with criteria in the Cochrane Handbook, version 5.1.0; these criteria include random sequence generation, allocation concealment, blinding of participants and personnel, blinding of outcome assessment, completeness of outcome data, selective reporting, and other sources of bias.^[15]^

### 7.3 Data synthesis

Differences between the intervention and control groups will be assessed. Mean differences (MDs) with 95% confidence intervals (CIs) will be used to measure the effects of treatment for continuous data. We will convert other forms of data into MDs. For outcome variables on different scales, we will use standard MDs (SMDs) with 95% CIs. For dichotomous data, we will present treatment effects as relative risks (RRs) with 95% CIs; other binary data will be converted into RRs.

All statistical analyses will be conducted using the Cochrane Collaboration's software program Review Manager (RevMan) version 5.3 for Windows (Copenhagen, The Nordic Cochrane Centre, the Cochrane Collaboration, 2012). We will contact the corresponding authors of studies with missing information to acquire and verify data whenever possible. As appropriate, we will pool data across studies to conduct a meta-analysis using fixed or random effects. We will use GRADEpro software from Cochrane Systematic Reviews to create a summary of findings table.

### 7.4 Unit of analysis issues

For crossover trials, data from the first treatment period will be used. For trials that assessed more than 1 control group, the primary analysis will combine data from each control group. Subgroup analyses of the control groups will be performed. Each patient will be counted only once in these analyses.

### 7.5 Addressing missing data

Intention-to-treat analyses that include all randomized patients will be performed. For patients with missing outcome data, last observation carry-forward analysis will be conducted. When individual patient data are initially unavailable, we will review the original source and/or published trial reports to obtain these data.

### 7.6 Assessment of heterogeneity

On the basis of our data analyses, we will use random- or fixed-effect models to conduct the meta-analysis. Chi-squared and *I*-squared tests will be used to evaluate the heterogeneity of the included studies, with *I*^2^ >50% indicative of high heterogeneity. When heterogeneity is observed, subgroup analyses will be conducted to explore the possible causes of this heterogeneity.^[16]^

### 7.7 Assessment of reporting biases

Funnel plots will be generated to detect reporting biases when a sufficient number of included studies (at least 10 trials) are available.^[17]^ However, because funnel plot asymmetries are not equivalent to publication biases, we will aim to determine possible reasons for any asymmetries in the included studies; potential causes of such asymmetries include small-study effects, poor methodological quality, and true heterogeneity.^[17,18]^

## 8 Discussion

Bojungikki-tang is a widely used herbal medicine in various Asian countries, including China (Bu-Zhong-Yi-Qi-Tang in Chinese), Japan (Hochu-ekki-to in Japanese), and Korea (Bojungikki-tang in Korean). Bojungikki-tang refers to a decoction to tonify the middle and augment Qi.^[19]^ This medicine consists of 8 herbal ingredients: *Astragalus membranaceus* BUNGE (6 g), *Panax ginseng* C. A. Meyer (4 g), *Atractylodes macrocephala* Koidzumi (4 g), *Glycyrrhiza uralensis* Fischer (4 g), *Angelica sinensis* (Oliv.) Diels (2 g), *Citrus unshiu* Markovicht (2 g), *Cimicifuga heracleifolia* Komarov (1.2 g), and *Bupleurum falcatum* Linne (1.2 g). Bojungikki-tang was first introduced in Dong Yuan Ten Medical Books, a medical text written by Li Dong-yuan in China during the Yuan Dynasty.^[20]^ It has been known as a herbal prescription for treating digestive disorders or conditions involving alternating cold and heat, such as malaria, for hundreds of years.^[20,21]^ In recent research, anti-inflammatory and immunoregulatory effects of Bojungikki-tang have been revealed in animal experiments^[22,23]^ and clinical trials.^[24,25]^ These effects will also be beneficial for alleviating allergic symptoms. All types of Bojungikki-tang formulations, such as decoctions, tablets, pills, and powders, will be considered for inclusion in this review. Even trials for which the assessed Bojungikki-tang involved a different ratio of ingredients and/or additional or missing herbs relative to the original Bojungikki-tang will be included if their tested Bojungikki-tang is similar to the original Bojungikkit-tang from the perspective of traditional East Asian medicine. There are several systematic reviews of herbal medicines; however, such reviews did not provide detailed intervention information. For each Bojungikki-tang variant included in this review, explanations of the variant's composition and ingredient ratio, type of formulation, and prescription based on the identification of patient patterns will be provided; the clear effects of Bojungikki-tang will be shown via meta-analyses. This systematic review will provide evidence for the use of herbal medicines in the treatment of AR.

## Supplementary Material

Supplemental Digital Content
