# Supplementary material for: Herbal medicine (Bojungikki-tang) for allergic rhinitis: A protocol for a systematic review of controlled trials
Source: Medicine (Baltimore). 2018 Jan 19;97(3):e9551. doi: 10.1097/MD.0000000000009551 (PMC5779745; doi:10.1097/MD.0000000000009551)
Supplement: Supplemental Digital Content [file medi-97-e9551-s001.docx]

**Supplementary 1. Search strategy for MEDLINE database**

| #1 | Search ("Rhinitis"[Mesh] OR "Rhinitis, Allergic"[Mesh] OR "Rhinitis, Allergic, Perennial"[Mesh] OR "Rhinitis, Allergic, Seasonal"[Mesh]) |
| --- | --- |
| #2 | Search ((("Rhinit*"[tw] OR "rhinopath* "[tw] OR "rhinosinusit*"[tw] OR rhinoconjunctivitis[tw] OR ozena*[tw] OR hayfever[tw] OR "hay fever"[tw] OR pollinosis[tw] OR pollenosis[tw] OR pollonosis[tw]))) OR ("Rhinitis"[Mesh] OR "Rhinitis, Allergic"[Mesh] OR "Rhinitis, Allergic, Perennial"[Mesh] OR "Rhinitis, Allergic, Seasonal"[Mesh]) |
| #3 | Search (#1 OR #2) |
| #4 | Search "Chinese medicine"[Mesh] |
| #5 | Search "Kampo medicine" |
| #6 | Search "Korean medicine"OR "Traditional Korean medicine" |
| #7 | Search ((Plant Extracts"[tiab]) OR "Herbal Medicine"[tiab] or "herbal*")) OR (("Plant Extracts"[Mesh]) OR "Herbal Medicine"[Mesh] |
| #8 | ‘Bojungikki-tang’ OR ‘Bu-Zhong-Yi-Qi-Tang’ OR ‘Hochu-ekki-to’ [All Fields] |
| #9 | #3 OR #4 OR #5 OR # 7 |
| #10 | #2 AND #8 |
